# Altered Serotonin, Dopamine and Norepinepherine Levels in 15q Duplication and Angelman Syndrome Mouse Models

**DOI:** 10.1371/journal.pone.0043030

**Published:** 2012-08-16

**Authors:** M. Febin Farook, Michael DeCuypere, Keith Hyland, Toru Takumi, Mark S. LeDoux, Lawrence T. Reiter

**Affiliations:** 1 Department of Neurology, UTHSC, Memphis, Tennessee, United States of America; 2 Department of Neurosurgery, UTHSC, Memphis, Tennessee, United States of America; 3 Medical Neurogenetics, LCC, Atlanta, Georgia, United States of America; 4 Hiroshima University, School of Medicine, Hiroshima, Japan; 5 Department of Anatomy and Neurobiology, UTHSC, Memphis, Tennessee, United States of America; 6 Department of Pediatrics, UTHSC, Memphis, Tennessee, United States of America; University of Toronto, Canada

## Abstract

Childhood neurodevelopmental disorders like Angelman syndrome and autism may be the result of underlying defects in neuronal plasticity and ongoing problems with synaptic signaling. Some of these defects may be due to abnormal monoamine levels in different regions of the brain. *Ube3a*, a gene that causes Angelman syndrome (AS) when maternally deleted and is associated with autism when maternally duplicated has recently been shown to regulate monoamine synthesis in the *Drosophila* brain. Therefore, we examined monoamine levels in striatum, ventral midbrain, frontal cerebral cortex, cerebellar cortex and hippocampus in *Ube3a* deficient and *Ube3a* duplication animals. We found that serotonin (5HT), a monoamine affected in autism, was elevated in the striatum and cortex of AS mice. Dopamine levels were almost uniformly elevated compared to control littermates in the striatum, midbrain and frontal cortex regardless of genotype in *Ube3a* deficient and *Ube3a* duplication animals. In the duplication 15q autism mouse model, paternal but not maternal duplication animals showed a decrease in 5HT levels when compared to their wild type littermates, in accordance with previously published data. However, maternal duplication animals show no significant changes in 5HT levels throughout the brain. These abnormal monoamine levels could be responsible for many of the behavioral abnormalities observed in both AS and autism, but further investigation is required to determine if any of these changes are purely dependent on Ube3a levels in the brain.

## Introduction

Angelman syndrome (AS) is a severe neuro-developmental disorder with a prevalence of approximately 1 in 15,000 individuals. Characteristic features of this syndrome include developmental delays, speech impairment, ataxia, frequent inappropriate laughter and in some cases abnormal EEG patterns, seizures and microcephaly [1]. The underlying genetic causes of AS are maternal deletion of the 15q11-q13 region [2], paternal uniparental disomy [3], imprinting defects [4] that control *UBE3A* gene expression, and maternally derived or inherited point mutations in the *UBE3A* gene [5].

Maternal duplications of the 15q11-q13 region are clearly associated with an autism phenotype [6], more broadly classified as Autism Spectrum Disorder (ASD). The major characteristic features of ASD include impairment in social interaction, verbal and non-verbal communication problems, rigid or repetitive behavior and restricted interests [7]. Paternal duplications of 15q usually do not result in a detectable phenotype [8]. However, in some studies paternal duplications have been associated with autistic behavior [9].

The underlying molecular defect in AS is a loss of expression of the human *UBE3A* gene within neurons [10]–[12]. *UBE3A* is a maternally expressed gene within neurons of multiple regions of the brain with highest expression in the hippocampus and cerebellum [13]. The *UBE3A* gene encodes a ubiquitin E3 ligase protein called E6-associated protein (E6-AP) that is responsible for targeting proteins for ubiquitination, in some cases leading to protein degradation by the ubiquitin proteasome system [10], [14]. Another less explored function of UBE3A is its ability to act as a co-activator for steroid hormone receptors [15], which may be more relevant to the current study since we recently showed that the *Drosophila* orthologue of UBE3A, called Dube3a, can regulate monoamine synthesis indirectly through the transcriptional co-activation of GTP cyclohydrolase I, the rate limiting enzyme in monoamine synthesis [16]. Proper regulation of neurotransmitters like dopamine (DA) and serotonin (5-hydroxytryptamine; 5HT) is essential for the normal functioning of synaptic plasticity. It is possible that changing the levels of neurotransmitter in the brain may be responsible for some of the clinical features of both AS and ASD. For example, the anxiety and hyperactivity associated with ASD can often be ameliorated by selective serotonin reuptake inhibitors (SSRIs) [17]. Levels of tetrahydrobiopterin (THB), an essential regulatory cofactor for monoamine synthesis, are altered in at least some autistic individuals [18] implying that defects in monoamine synthesis could be associated with ASD phenotypes. Finally, 5HT levels have been shown to be higher in the platelets of individuals with ASD than controls [19], [20].

To date, several AS mouse models have been generated with various types of mutations that resemble both chromosomal and point mutations. Initially, Jiang *et al.* generated a *Ube3a* deficient mouse model by knockout of exon 2 of the *Ube3a* gene [21]. Mice that carry this mutation from the mother exhibit ataxia, epilepsy and extended EEG polyspikes [21]–[23]. Maternally deficient *Ube3a* mice (*Ube3a^(M^* ^−/P+)^) show learning and memory defects [21] as well as defects in experience dependent synaptic plasticity [24]. Our group has also identified motor abnormalities in fluid licking patterns in *Ube3a* deficient mice [25], a phenotype known to be regulated by the cerebellum [26]. These phenotypes may be due to neurochemical changes in the brain since overall morphology of the cerebellum appears normal [25]. Paternally deficient mice (*Ube3a^(M^* ^+/P−)^) failed to show any of these characteristics [21], [24], [25].

A murine model for 15q interstitial duplication syndrome was recently created by Nakatani *et al.* using chromosomal engineering methods to replicate the 15q duplication in a syntenic region of the mouse genome on chromosome 7p [27]. Since several genes in the duplicated region are imprinted including *Ube3a*, which is maternally expressed in neurons, the duplication is classified as either paternal or maternal duplication based on the origin of the duplicated allele. Somewhat surprising was the finding that paternal duplication animals showed impaired social interaction, as opposed to maternal duplication mice, when compared to wild type littermates [27]. This finding is somewhat puzzling since maternal, but not paternal, duplications of 15q are associated with autism in humans [8]. Subsequent studies using a mouse that has multiple genomic copies of the *Ube3a* gene, thus forcing increased expression of the maternally imprinted *Ube3a* gene artificially, showed that indeed elevated *Ube3a* expression in the mouse brain is associated with autism like behaviors [28].

The aim of this study was to determine if changing *Ube3a* levels has a direct effect on monoamine levels in different brain regions by using both *Ube3a* deletion and duplication mouse models. Here we show that *Ube3a* may be involved in regulating some, but not all of these monoamines thereby influencing synaptic plasticity in the brain. These regional changes in monoamine levels could explain some of the behavioral abnormalities found in both mice and humans when *Ube3a* levels are decreased or elevated due to chromosomal abnormalities.

## Materials and Methods

### Animals Husbandry

All mice were maintained on a C57BL/6J background and all experiments performed in accordance with guidelines approved by the University of Tennessee Health Science Center Institutional Animal Care and Use Committee (IACUC). The generation and maintenance of *Ube3a* deficient mice was done as described previously [21]. Paternal and maternal mouse 7p duplication animals were generated as described [27]. Animals were maintained in 12 hour light/dark cycles and had access to food and water *ad libidum.* Mice between the ages of 12–15 weeks old were used for all experiments. Wild type littermates were used as controls. For all experiments, six animals were used for each genotype (n = 6), unless otherwise indicated.

### Chemicals

HPLC-grade DA, 3,4-dihydroxyphenylacetic acid (DOPAC), epinephrine (E), ethylenediaminetetraacetic acid (EDTA), homovanillic acid (HVA), 5-hydroxyindole-3-acetic acid (HIAA), norepinephrine (NE), *ortho*-phosphoric acid, perchloric acid, 5-hydroxytryptamine (5HT), sodium bisulfite, triethylamine (TEA) and 3,4-dihydroxybenzylamine (DHBA) were purchased from Sigma-Aldrich (St. Louis, MO, USA). Sodium octylsulphonate (SOS) and monobasic anhydrous sodium dihydrogen phosphate used in mobile phase preparation were purchased from Fluka Chemie (Buchs, Switzerland). HPLC grade water and acetonitrile were obtained from Fisher Scientific (Hampton, NH, USA).

### Sample Preparation

Mice were briefly anesthetized with aerosolized isoflurane and fresh brain samples were obtained and partitioned using a dissecting microscope. Tissue samples (approximately 0.05–0.1 g) of striatum, ventral midbrain, frontal cerebral cortex, cerebellar cortex and hippocampus were weighed and then homogenized in 100 µl of ice-cold dissolution buffer (0.1 M perchloric acid, 0.1 mM sodium bisulfite, and 0.1 mM EDTA) per 10 mg wet weight. All homogenates were centrifuged at 20,000 *g* for 25 min at 4°C. Supernatants were filtered through 0.22 µm pore size polyvinylidene fluoride (PVDF) syringe-driven membrane filters (Millipore Corp., Bedford, MA, USA) and immediately frozen and stored at −80°C until the time of analysis.

### High-Performance Liquid Chromatography with Electrochemical Detection (HPLC-EC)

Primary stock standard solutions were prepared by dissolving 10 mg of DA, DOPAC, E, HIAA, 5HT, HVA and NE in 25 ml of dissolution buffer. These concentrates were then divided into 1 ml aliquots, frozen, stored at −80°C and thawed prior to use at 4°C. Working standards in the ηM range were freshly prepared prior to each assay. Standard curves employed for analyte quantitation were generated with an internal standard 3,4-dihydroxybenzylamine (DHBA). The relationship between concentration and relative response was linear over two orders of magnitude (sample correlation coefficient >0.98), with an overall electrochemical sensitivity of 0.001 mA. The detection limit for all analyses was 20 ρg per injection.

### Mobile-Phase Preparation

A stock buffer solution containing 75 mM monobasic sodium dihydrogen phosphate, 2 mM SOS, 25 µM EDTA and 100 µl of TEA was prepared in 1800 ml of HPLC grade water. To prepare the mobile phase, this solution was then mixed with 200 ml of HPLC grade acetonitrile and buffered to pH 3.0 using concentrated *ortho*-phosphoric acid. The mobile phase was stored at room temperature for no more than 2 days, filtered through a 0.20 µm pore size white nylon filter membrane (Millipore Corp.) and degassed under vacuum for 30 min prior to use.

### Liquid Chromatography

HPLC-EC analysis was performed with an ESA Model 5600A CoulArray® system (ESA Inc., Chemlsford, MA, USA), equipped with Shimadzu Model DGU-14A on-line degassing unit (Shimadzu Scientific Instruments, Columbia, MD, USA), an ESA Model 582 pump and an ESA Model 542 refrigerated autosampler. The detection system consisted of three coulometric array modules, each containing four electrochemical detector cells. Electrode potentials were selected over the range of 0 to +700 mV, with a 50 mV increment against palladium electrodes. Chromatographic separation was achieved by auto-injecting 30 µl sample aliquots at 5°C onto a MetaChem Intersil (MetaChem Technologies, Torrance, CA, USA) reversed-phase C_18_ column (5 µm particle size, 250×4.6 mm I.D) with an ESA Hypersil C_18_ pre-column (5 µm particle size, 7.5×4.6 mm I.D.). A mobile phase flow rate of 1.25 ml/min and analysis time of 45 min was used for all experiments. System control and data acquisition/processing were performed using ESA CoulArray software (version 1.02). All samples were processed in technical triplicate with median values used for mean calculations.

### BH_4_ Measurements

BH_4_ measurement was done as described previously [20], [29]. Briefly, brain tissue samples were homogenized to 25% w/v in ice cold 0.1 mol/L perchloric acid containing dithioerythritol (DTE) (6.5 mmol/L) and diethylenetriaminepentaacetic acid (DETAPAC) (2.5 mmol/L). The homogenates were then centrifuged at 15,000 g for 5 minutes and the supernatants were collected. BH_4_ analysis was performed using supernatants via reverse-phase HPLC–EC as described by Howells and Hyland [29].

### Quantitative Western Blot Analysis

Tissue samples (approximately 0.05–0.1 g) of cerebellum and hippocampus were weighed, homogenized and proteins extracted in radioimmunoprecipitation assay (RIPA) buffer. The soluble proteins were then resolved on a NuPage 4–12% Bis-Tris 1.5 mm Gel using MOPS buffer (Invitorgen, Carlsbad, CA) and transferred to an Immobilon-FL PVDF membrane (Millipore). The membrane was blocked with 5% milk, 3% BSA and 0.1% Tween-20 in Phosphate Buffered Saline (PBS). α-E6-AP (Sigma-Aldrich) and α-GAPDH (Imgenex) primary antibodies were used at a concentration of 1∶1000 and 1∶2500, respectively. Infrared (IR) labeled secondary antibodies (α-Rabbit 800 and α-Goat 680) from Li-Cor (Lincoln,NE) were used at a dilution of 1∶10,000. The blot was imaged and analyzed using the Odyssey Infrared Imaging System (Li-Cor, Linclon, NE). Lanes were normalized using signals from GAPDH as the reference channel. Percentage change was calculated by dividing the normalized intensity value of each sample to that of wild type control lane intensity for E6-AP.

### Data Analysis

Statistical analysis was conducted by using Graphpad Prism. Data were analyzed by unpaired *t*-test and one way ANOVA. The criterion for significance was set at p≤0.05.

## Results

### Ube3a Expression Changes are Restricted to the Maternal Allele in Both the Ube3a Deficient and 7p Duplication Mouse Models

**Figure 1:**
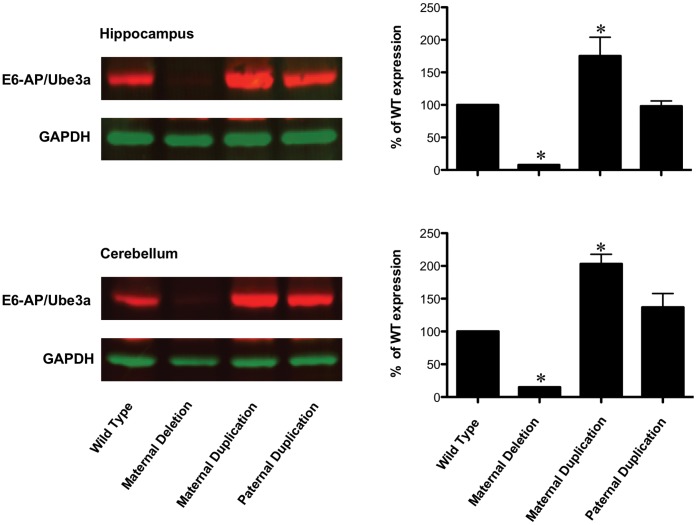
E6-AP/Ube3a expression levels are decreased in *Ube3a* deficient mice in the hippocampus and cerebellum and increased in maternal 7p duplication animals. Ube3a protein shows significant reduction (>80%; p≤0.05 and n = 3) in *Ube3a* deficient mice and a 75% increase in 7p maternal duplication animals (p≤0.05 and n = 3). A slight increase in Ube3a expression was also observed in 7p paternal duplication animals compared to wild type animals but did not reach significance. *p≤0.05 compared to wild type.

Since our current hypothesis is that Ube3a protein levels alone will have downstream effects on monoamine synthesis, we needed to establish in a quantitative manner if both mouse models used in this study conform to the current imprinting paradigm for *Ube3a* expression. Specifically, we were interested in establishing that Ube3a levels are significantly elevated in the 7p duplication animals only when the duplicated allele is inherited maternally. In fact, our quantitative IR Western blot results indicate that when a *Ube3a* loss of function mutation generated by Jiang *et al.* is maternally inherited, there is a >80% decrease in Ube3a protein in both the cerebellum and hippocampus. Similarly, there is >75% increase in Ube3a protein in the maternal 7p duplication animals but not the paternal duplication animals in the cerebellum and hippocampus (**Figure 1**). These results are consistent with two previous publications [21], [27], thus making the 7p duplication mouse model a valid model for maternal duplication 15q autism where Ube3a levels are elevated in neurons [30].

### Regulation of Monoamines is Only Partially Dependent on Ube3a Levels in the Brain

**Figure 2:**
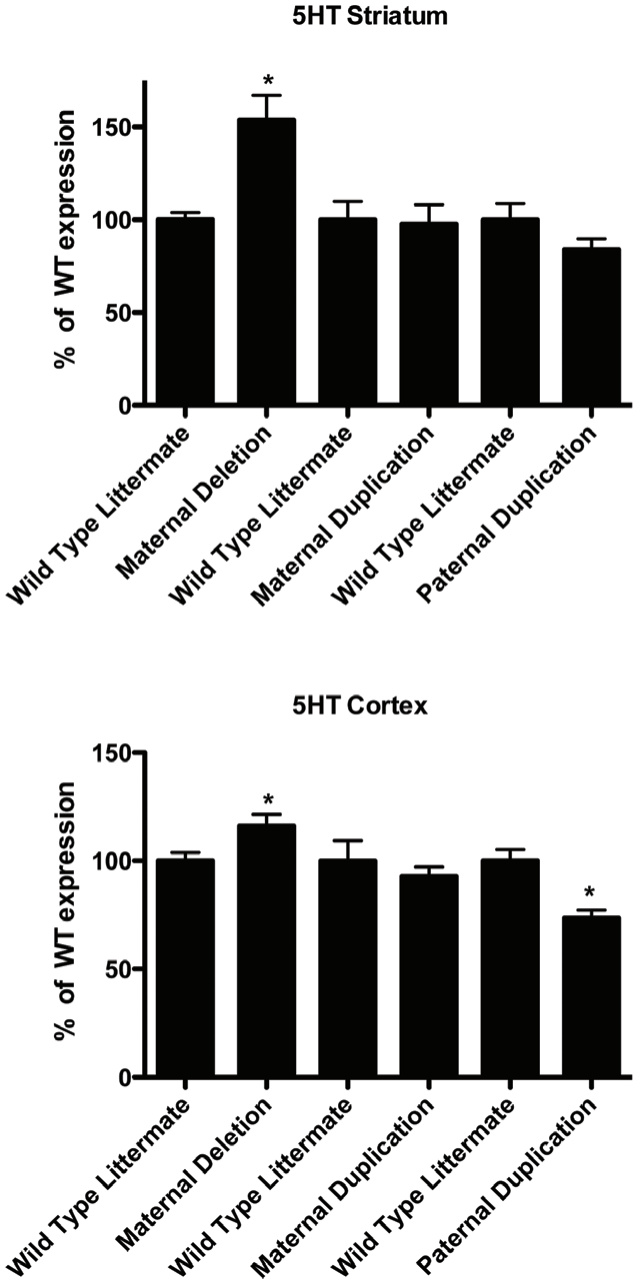
Serotonin (5HT) levels are elevated in *Ube3a* deficient mice in the striatum and cortex and decreased in paternal 7p duplication animals. No significant change in 5HT was seen in *Ube3a* duplication animals in the striatum. Maternal 7p duplication animals did not show any significant changes in 5HT in either striatum or frontal cortex. In all cases, 5HT levels for each genotype were compared to wild type littermates from each cross (100%). Significant differences were found (p≤0.05 and n = 6) for all groups. *p≤0.05 compared to wild type littermate.

We quantified the levels of monoamines and monoamine precursors as well as co-factors needed for monoamine synthesis in 5 regions of the brain (cerebellum, frontal cerebral cortex, striatum, hippocampus and ventral midbrain) in both *Ube3a* deficient and *Ube3a* duplication animals (**Table S1**). In maternal *Ube3a* deficient mice, 5HT levels were significantly increased in the frontal cortex and striatum compared to wild type littermates. In paternal duplication animals, 5HT levels decreased in both of these regions significantly compared to their wild type littermates, while in maternal duplication animals there were no significant changes in 5HT levels (**Figure 2**). HIAA, a major metabolite of 5HT, showed a similar trend in both cortical and striatal regions of the brain. In addition, 5HT levels also decreased significantly in hippocampus and midbrain regions in paternal duplication animals (**Figure 2**). Our results indicate that a decrease in Ube3a levels increases brain 5HT, while paternal duplication of the gene region containing *Ube3a* also results in a decrease in 5HT. Therefore this effect is not exclusively due to *Ube3a* expression levels.

**Figure 3:**
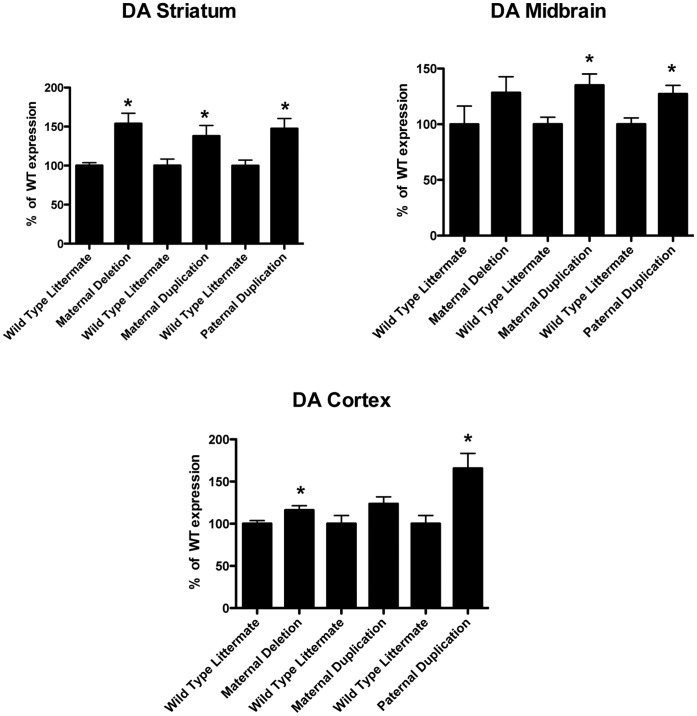
Dopamine (DA) levels are increased in striatum, midbrain and frontal cortex under conditions of increased and decreased *Ube3a* expression. A significant increase was detected in DA levels in *Ube3a* deficient mice in both striatum and frontal cortex (p≤0.05) and a clear trend was detected in midbrain that did not reach significance. Animals with a 7p duplication encompassing the *Ube3a* gene showed a significant increase in DA levels in all three regions when the duplication was paternally inherited, but only showed a significant increase in striatum and frontal cortex when maternally inherited, although a trend towards increased DA is detected here as well. In all cases, DA levels for each genotype were compared to wild type littermates from each cross (100%). Significant differences were found at p≤0.05 for all groups (n = 6). * p≤0.05 compared to wild type littermate.

Somewhat surprising was our finding that DA levels increased significantly in both deletion and duplication animals in both the striatum and midbrain (**Figure 3**). In the frontal cortex, DA levels increased significantly in both maternal deletion and paternal duplication animals as compared to controls. No significant changes were observed for DA in maternal duplication animals in the cortex, but a significant increase in DA levels was seen in cerebellum (**Table S1**). Thus, regional DA levels appear to be sensitive to changes in Ube3a expression but are also affected by the expression of other genes in the duplicated region.

In *Ube3a* deficient animals, DOPAC levels increased significantly in the striatum and midbrain as compared to controls. In maternal duplication animals, DOPAC levels increased in striatum and in paternal duplication animals DOPAC levels increased in cerebellum, midbrain and frontal cortex (**Table S1**). Thus, there is some discrepancy about how changes in *Ube3a* affect DOPAC levels and these results may be an artifact of the experimental procedures or may reflect the rapid metabolism of DA in these regions due to supra-physiologic - DA levels (**Figure 3**).

**Figure 4:**
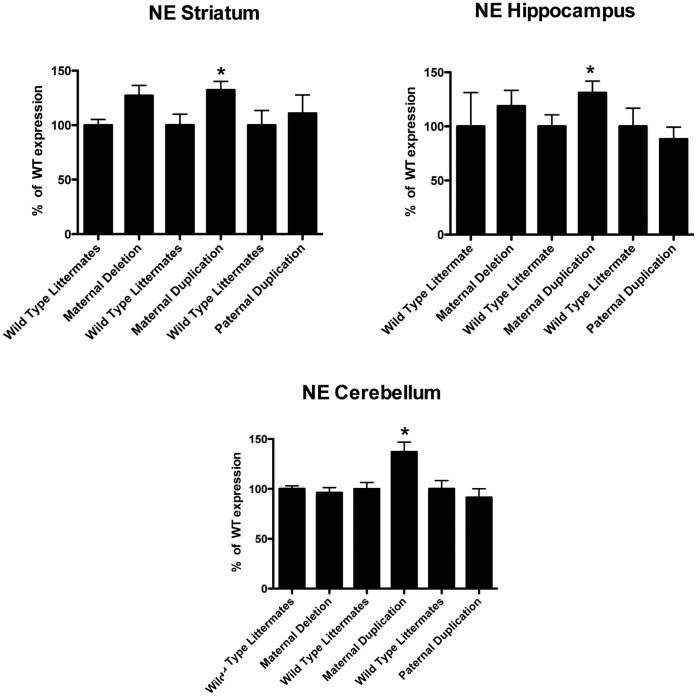
Maternal duplication 7p animals show a significant increase in norepinepherine levels in striatum, hippocampus and cerebellum. Maternal duplication animals that express higher levels of Ube3a than their wild type littermates also showed a significant increase in norepinepherine levels (unpaired *t*-test, p≤0.05). Elevated norepinepherine levels were also detected in *Ube3a* deficient animals in the striatum, but not other regions of the brain. Paternal duplication animals that do not demonstrate elevated levels of Ube3a did not show any changes in norepinepherine in the brain regions tested. In all cases, norepinepherine levels for each genotype were compared to wild type littermates from each cross (100%). Significant differences were found at p≤0.05 for all groups (n = 6). *p≤0.05 compared to wild type littermate.

Epinephrine increased significantly in the midbrain of maternal deletion animals, but in maternal duplication animals epinephrine showed an increase in the striatum and frontal cortex, not midbrain. Paternal duplication animals showed a significant increase in epinephrine in the cerebellum and cortex. Thus, while Ube3a can regulate DA in the midbrain, duplication of the region results in a significant increase in the frontal cortex. Norepinephrine changed significantly in maternal duplication animals within the striatal, hippocampal and cerebellar regions (**Figure 4**). Thus, in these regions of the brain, norepinepherine appears to respond to increased Ube3a levels, while decreased amounts of Ube3a only affected NE levels in the midbrain (**Table S1**).

Finally, we wanted to determine if monoamine levels were regulated in the mouse brain by Ube3a by the same mechanism we established in the fly brain. In both flies and mice GTP cyclohydrolase I produces a key co-factor for monoamine synthesis, tetrahydrobiopterin (BH_4_). We found that increasing or decreasing Dube3a levels in the fly brain resulted in increased or decreased BH_4_ levels and DA levels, respectively [16]. When we measured BH_4_ levels in the cerebellum, frontal cortex, striatum, hippocampus and midbrain of *Ube3a* deficient and duplication animals we found that BH_4_ levels did not change significantly in any region tested across all genotypes (**Table S1**). We did, however, notice a trend in *Ube3a* deficient animals suggesting that BH_4_ levels may decrease slightly when Ube3a levels decrease, but these changes were not consistent with the detection of significant increases in monoamines levels in *Ube3a* deficient animals (**Figures 2** **and** **3**).

## Discussion

Although mouse models have been generated for both loss of *Ube3a* and over-expression of *Ube3a* (which approximate at least some of the features of both Angelman syndrome and 15q duplication autism) questions remain about how changes in *Ube3a* levels or other genes in the duplicated/deleted region are involved in neuronal function and behavior. For example, *Ube3a* deficient mice have learning and memory defects and motor abnormalities [21], [25] that include abnormal cerebellar controlled licking patterns [25], all of which can not be attributed to obvious cellular changes in the brain. In fact, some of the characteristic features of AS like social seeking behavior and hyperactivity are not found in the *Ube3a* maternal deletion mouse [31]. In this study, we investigated monoamine concentrations in different regions of the mouse brain in order to localize neurochemical changes that may account for some of these behaviors and aberrant neural activities.

We found that maternal deficient *Ube3a* mice showed a significant increase in the amount of 5HT in the striatum and cortex when compared to wild type littermates and that paternal duplications of the *Ube3a* gene region, which do not cause increased Ube3a protein levels in the brain (**Figure 1**), resulted in decreased 5HT levels as previously observed [32]. Maternal duplication animals did not show any significant change in 5HT levels when compared to their wild type littermates. This is consistent with previous studies of 5HT in these animals [32], but indicates that these changes in 5HT, although counter to those observed in *Ube3a* maternal deficient animals, are not dependent on *Ube3a* expression levels. HIAA, a major metabolite of 5HT, also showed a similar trend in these three genotypes, but did not reach significance (**Table S1**). The involvement of the serotonin system in autism has been well studied and reduced serotonin transporter (SERT) binding capacity in the frontal cortex has been found in autistic children [33]. In fact, SSRIs can modulate anxiety and hyperactivity associated with autism [17], suggesting that an underlying defect in the ability to regulate 5HT levels in the brain may underscore autism in general. In mice, SSRIs can modify behavioral abnormalities like decreased exploratory activity, as well as increased anxiety and depression during early developmental stages [34], [35]. Thus, identifying direct correlates between the mouse model and human phenotypes may prove difficult.

Elevated levels of 5HT in the blood are often a hallmark of autism [36]. Studies in 5HT_1A_ receptor knockout mice have shown that blood 5HT levels were normal at birth, but after two weeks there was a reduction of blood 5HT levels in these animals, which eventually returned to normal levels in 5HT_1A_ knockout adults [37]. These knockout mice showed a reduction in brain 5HT levels at 3 days after birth, which resolve to normal levels in as they approach adulthood. These studies suggests that the 5HT receptors can have an effect on 5HT levels during embryonic and post-natal development, but play a lesser role in modulating adult 5HT levels in mice. Nevertheless, autism-like phenotypes are found in mice defective in certain 5HT receptors like 5HT_1A_ 5HT_2C_ and 5HT_7_ [38]–[40]. The 15q11.2–13 region, duplicated in some cases of autism and deleted in ∼70% of all AS patients, contains the *SNORD-115* gene, called *MBII-52* in mice, a small nucleolar RNA which regulates the 5HT_2C_ receptor and is expressed at two-fold higher levels in paternal duplication animals [27]. Since Ube3a levels are comparable to wild type animals in paternal duplication mice, so this effect of *MBII-52* duplication on the 5HT_2C_ receptor does not appear to be Ube3a dependent and warrants further investigation. The 5HT transporter (SERT), which is essential for the uptake of biogenic amine into blood platelets, can influence 5HT levels through increased uptake of 5HT or higher than normal binding levels [41]–[43]. In fact, variants in the gene encoding the 5HT transporter (*SLC6A4*) can cause an ASD phenotype [44], [45]. Recently a mouse was constructed containing a high 5HT affinity mutation in SERT that has been associated with autism in humans (Ala56) [46]. This mouse has pronounced hyperserotonemia, but only mild impairment in social communication and interaction [46]. Although increased levels of 5HT were found in the blood platelets of these mice, no changes in 5HT levels were found in the brain. Still the mice showed partial expressivity for ASD as indicated by decreased ultrasonic vocalizations in pups and reduced social interactions with other mice versus inanimate objects. The authors suggest that these effects could be due to the reduced availability of 5HT, due to excessive 5HT clearance during early stages of brain development. The mouse model for autism we examined in this study (paternal duplication 7p animals) also shows decreased exploratory activity along with reduced 5HT levels in various brain regions [32]. Interestingly, in our studies, the Angelman mouse model also has similar phenotypes like reduced exploratory activity and impaired social interactions [31], but these mice have elevated levels of 5HT in various brain regions. Our results, in combination with those from the SERT Ala56 mice, suggest that decreasing levels of 5HT in the brain produces autism-like behaviors while elevated levels of 5HT may be associated with more severe intellectual disability and possibly even seizure phenotypes that are present in humans with AS and the AS mouse model.

5HT may also be a key player in synaptic development and maturation. Altered 5HT signaling is responsible for many neurodevelopmental disorders including depression, anxiety, obsessive-compulsive disorder, schizophrenia and sudden infant death syndrome. The prevailing hypothesis for the role of 5HT neurons in mental health disorders is that the genetic determinants and environmental factors influencing them can produce abnormal levels of 5HT, which in turn effect the small population of 5HT neurons disrupting the normal development of the synapses [47]. One third of autistic children have increased levels of 5HT in their blood but in adults these changes are not so obvious [19], [48]. SSRIs can modulate episodes of paroxysmal laughter in patients suffering from pathological laughter due to stroke, neurodegenerative disease, or brain injury [49], [50]. This effect may also be observed in otherwise healthy individuals [51]. Although this has not been specifically tested in Angelman syndrome patients, SSRIs have been shown to decrease anxiety, impulsivity and hyperactivity in this population [52]. Changes in 5HT synthesis capacity have also been observed in ASD individuals, particularly in children, where 5HT synthesis was found to be disrupted in children with autism [53]. We recently observed that fly Ube3a (Dube3a) can regulate BH_4_ through the transcriptional co-activation of GTP cyclohydrolase I, a rate limiting enzyme for monoamine biosynthesis [16]. In the current study, however, we were unable to detect significant changes in BH_4_ levels in any of the genotypes. In addition, gene expression studies in these brain regions did not indicate significant changes in GTP cyclohydrolase I transcript levels in any of the genotypes tested (data not shown). However, slightly decreased levels of BH_4_ were detected in striatum, hippocampus, midbrain and frontal cortex in *Ube3a* deficient mice (**Table S1**). Since elevated monoamine levels were found in several regions of the brain in all genotypes tested, it could be possible that BH_4_ levels were negatively regulated by the downstream increase in monoamine levels, thus normal BH_4_ levels were detected.

Two additional characteristic features of Angelman syndrome in humans are social seeking behavior and hyperactivity. However, these characteristics are not found in the Angelman syndrome mouse models. In fact, these animals have reduced social behavior and reduced exploratory behavior [31]. Increasing 5HT levels in rodent models through the inhibition of normal 5HT re-uptake is known to result in reduced social and exploratory behaviors consistent with our findings of elevated 5HT in the AS mouse model [40]. It may well be that loss of *UBE3A* in humans results in some AS associated phenotypes due to elevated 5HT levels since DA depletion therapy with reserpine was able to ameliorate uncontrolled myoclonus in at least one AS patient [54].

Although *Ube3a* is the causative gene in Angelman syndrome and may be contributing to 15q duplication autism, many of the features observed in the *Ube3a* deficient AS mouse model are not the opposite of what is observed in the *Ube3a* duplication autism mouse models. In other words, many of the phenotypes attributed to decreased *Ube3a* levels are not opposite when *Ube3a* levels are increased. Glutamatergic synaptic defects observed in a recent mouse model for autism due to increased Ube3a alone do not entirely reflect the inverse effects of lower levels observed in *Ube3a* deficient animals [28]. Increased Ube3a reduces mini excitatory post-synaptic current (mEPSC) frequency in both *Ube3a* deletion and duplication animals in the visual cortex and somatosensory structures [24], [28]. Greer *et al*. attributed the effects of reduced glutamatergic synaptic transmission, evolved AMPA/NMDA current ratio and reduced mEPSC amplitude in hippocampal CA1 pyramidal neurons to the degradation of ARC (activity-related cytoskeleton-associated protein), a putative Ube3a ubiquitination target known to regulate AMPA receptor trafficking [55]. Smith *et al*. confirmed that increasing Ube3a levels reduced ARC protein levels but did not reverse the electrophysiological effects observed in *Ube3a* deficient animals [28]. Studies also show that decreased Ube3a reduces dendritic spine density, which may explain the observed reduced mEPSC frequency reported in the visual cortex of these animals. However, increasing Ube3a levels does not change dentritic spine density, yet still results in a reduction of mEPSC frequency [13], [24], [28]. In short, decreasing or increasing Ube3a levels alone may contribute in different ways in both Angelman syndrome and 15q duplication autism phenotypes rather than resulting in clear diametrically opposed affects.

In our studies, DA levels were significantly elevated in both deletion and duplication animals in the striatum and midbrain. In the cortex, DA levels increased in both maternal deletion and paternal duplication animals. Increased DA levels have been found in *Ube3a* paternal duplication animals during developmental stages [32]. These increased DA levels may be influencing the reduced exploratory behavior and hypoactivity observed in both of these animal models [31], [32].

In summary, this study demonstrates that 5HT levels increase in maternal deficient *Ube3a* mice that have reduced Ube3a levels in neurons but there was no significant decrease in 5HT levels in *Ube3a* maternal duplication animals when Ube3a levels increased. However, the behavioral phenotypes seen in these two different animal models are quite similar indicating that a different mechanism may be involved in each, independent of or in addition to Ube3a regulation of 5HT levels. Increased DA levels can also influence various developmental processes in early brain developmental stages, so the changes in DA found in both animal models could have contributed to the phenotypes observed. Our study suggests that Ube3a levels can influence 5HT levels indirectly in the brain yet a role for Ube3a in monoamine synthesis in mammals remains unclear. Since Ube3a regulates a set of primarily unknown substrates it is possible that altered signaling, feedback regulation or changes in 5HT receptor levels or function could explain the changes in monoamine levels we observed. The changes in monoamine levels observed here might result from a number of defects where Ube3a is only partly responsible or where Ube3a levels must maintain a critical level during a particular developmental stage.

## Supporting Information

Table S1 **Monoamine and BH4 levels in different tissue.** 5HT-5-hydroxytryptamine (serotonin), DA -dopamine, DOPAC-3,4-dihydroxyphenylacetic acid, E-epinephrine, HIAA-5-hydroxyindoleacetic acid, HVA -homovanillic acid, NE-norepinephrine, BH4-tetrahydrobiopterin. Values are means (n = 6).
(PDF)
