# Supplementary material for: Altered Serotonin, Dopamine and Norepinepherine Levels in 15q Duplication and Angelman Syndrome Mouse Models
Source: PLoS One. 2012 Aug 16;7(8):e43030. doi: 10.1371/journal.pone.0043030 (PMC3420863; doi:10.1371/journal.pone.0043030)
Supplement: Table S1 — Monoamine and BH4 levels in different tissue. 5HT-5-hydroxytryptamine (serotonin), DA -dopamine, DOPAC-3,4-dihydroxyphenylacetic acid, E-epinephrine, HIAA-5-hydroxyindoleacetic acid, HVA -homovanillic acid, NE-norepinephrine, BH4-tetrahydrobiopterin. Values are means (n = 6). (PDF) [file pone.0043030.s001.pdf]

***Ube3a* Maternal deletion animals**

Region	Genotype	5HT	DA	DOPAC	E	HIAA	HVA	NE	BH_4
**Striatum**	WT	106.34 ± 9.97	10742.62 ± 1115.34	218.41 ± 74.55	40.56 ± 7.85	23.51 ± 4.82	42.22 ± 6.99	28.35 ± 6.17	660.05 ± 122.49
	Ube3a$^{(M-/P+)}$	183.89 ± 15.33	17495.75 ± 2516.33	338.88 ± 47.83	43.425 ± 5.96	85.36 ± 7.12	58.51 ± 10.65	36.02 ± 6.49	492.13 ± 95.14
**Hippocampus**	WT	24.42 ± 10.73	117.06 ± 5.51	325.40 ± 33.54	18.27 ± 7.11	8.74 ± 3.84	27.04 ± 9.31	21.82 ± 16.70	215.25 ± 20.32
	Ube3a$^{(M-/P+)}$	16.82 ± 4.36	115.25 ± 6.37	338.48 ± 21.51	19.54 ± 9.34	6.74 ± 3.36	25.80 ± 6.52	27.57 ± 9.87	183.92 ± 22.60
**Cerebelllum**	WT	44.52 ± 24.97	14.94 ± 6.61	308.19 ± 50.22	11.71 ± 4.95	14.77 ± 8.28	16.44 ± 5.12	324.79 ± 24.76	236.7 ± 21.57
	Ube3a$^{(M-/P+)}$	33.18 ± 15.24	16.68 ± 3.90	292.97 ± 17.67	16.36 ± 5.43	13.70 ± 6.29	17.51 ± 5.16	312.44 ± 2.49	227.18 ± 86.83
**Midbrain**	WT	50.03 ± 20.05	253.88 ± 44.76	307.08 ± 53.33	8.87 ± 4.18	16.70 ± 6.69	40.96 ± 9.98	18.00 ± 6.36	490.25 ± 71.83
	Ube3a$^{(M-/P+)}$	64.20 ± 17.67	332.98 ± 28.17	406.84 ± 49.36	17.40 ± 6.45	19.23 ± 6.16	46.77 ± 8.82	37.32 ± 10.04	400.82 ± 63.37
**Cortex**	WT	653.63 ± 61.37	385.15 ± 15.55	465.53 ± 80.99	211.64 ± 22.27	204.30 ± 15.33	168.58 ± 29.80	159.03 ± 16.50	318.23 ± 48.51
	Ube3a$^{(M-/P+)}$	802.48 ± 57.68	423.91 ± 18.79	480.10 ± 23.08	213.70 ± 19.18	238.25 ± 19.08	246.55 ± 41.76	167.32 ± 22.79	240 ± 60.22

***Ube3a* Maternal duplication animals**

Region	Genotype	5HT	DA	DOPAC	E	HIAA	HVA	NE	BH_4
**Striatum**	WT	310.58 ± 75.58	**16584.72 ± 3411.70**	183.68 ± 41.99	**25.43 ± 10.09**	47.76 ± 14.00	159.33 ± 36.50	**166.75 ± 41.26**	270.7 ± 168.65
	Ube3a$^{(Mdup/+)}$	303.80 ± 78.76	**22873.16 ± 5500.82**	363.99 ± 134.40	**53.22 ± 14.21**	28.19 ± 15.06	187.57 ± 17.95	**220.47 ± 32.36**	254.97 ± 148.53
**Hippocampus**	WT	19.47 ± 6.25	103.01 ± 23.82	90.00 ± 27.97	62.01 ± 36.37	23.95 ± 6.11	25.67 ± 5.80	**74.49 ± 19.50**	124.2 ± 60.50
	Ube3a$^{(Mdup/+)}$	16.59 ± 3.23	118.18 ± 19.94	102.87 ± 44.19	75.25 ± 25.78	26.96 ± 8.47	21.83 ± 5.83	**97.65 ± 19.77**	124.1 ± 66.59
**Cerebelllum**	WT	28.89 ± 16.51	**13.20 ± 4.82**	52.70 ± 23.74	42.19 ± 14.27	18.68 ± 5.88	**24.58 ± 8.17**	**142.38 ± 22.49**	127.02 ± 59.36
	Ube3a$^{(Mdup/+)}$	35.43 ± 13.25	**25.92 ± 6.30**	61.02 ± 10.92	44.73 ± 11.11	18.13 ± 6.67	**37.34 ± 5.61**	**195.09 ± 34.75**	138.8 ± 69.24
**Midbrain**	WT	76.30 ± 15.82	**254.76 ± 39.42**	**161.81 ± 18.63**	20.35 ± 6.39	19.5 ± 8.57	**47.71 ± 7.75**	249.25 ± 56.44	234.95 ± 167.91
	Ube3a$^{(Mdup/+)}$	65.56 ± 13.92	**344.17 ± 62.96**	**184.78 ± 32.77**	22.45 ± 7.86	21.44 ± 6.69	**63.92 ± 14.65**	240.54 ± 58.10	294.72 ± 151.34
**Cortex**	WT	758.22 ± 173.89	482.96 ± 116.03	287.75 ± 34.10	**126.60 ± 27.82**	288.46 ± 27.08	271.64 ± 51.47	278.76 ± 53.89	153.62 ± 100.98
	Ube3a$^{(Mdup/+)}$	704.08 ± 80.09	597.15 ± 97.16	316.11 ± 80.91	**202.62 ± 18.48**	275.17 ± 39.55	300.34 ± 66.68	266.2 ± 79.28	171.25 ± 116.71

***Ube3a* Paternal duplication animals**

Region	Genotype	5HT	DA	DOPAC	E	HIAA	HVA	NE	BH_4
**Striatum**	WT	**336.62 ± 71.85**	**17631.15 ± 3033.51**	314.51 ± 35.27	126.09 ± 20.48	**124.09 ± 18.95**	**187.25 ± 51.22**	29.68 ± 9.74	868.00 ± 198.27
	Ube3a$^{(+/Pdup)}$	**282.99 ± 46.75**	**25981.75 ± 5628.18**	286.94 ± 47.10	131.53 ± 13.95	**86.65 ± 26.93**	**262.56 ± 88.39**	32.90 ± 12.27	834.50 ± 103.94
**Hippocampus**	WT	**195.74 ± 31.23**	128.18 ± 12.15	273.49 ± 44.78	100.15 ± 25.23	**59.72 ± 24.70**	27.89 ± 13.52	21.43 ± 8.80	390.33 ± 195.97
	Ube3a$^{(+/Pdup)}$	**124.49 ± 14.78**	122.70 ± 10.05	292.78 ± 26.16	80.58 ± 24.31	**23.06 ± 11.21**	32.11 ± 22.71	18.90 ± 5.81	269.33 ± 105.53
**Cerebelllum**	WT	67.55 ± 11.77	16.36 ± 7.64	**160.20 ± 41.30**	**100.33 ± 33.48**	37.76 ± 8.42	16.15 ± 4.44	259.49 ± 52.92	251.33 ± 16.80
	Ube3a$^{(+/Pdup)}$	58.39 ± 16.84	17.84 ± 6.41	**250.75 ± 69.59**	**172.11 ± 36.71**	25.76 ± 6.41	20.27 ± 6.43	237.13 ± 55.61	339.00 ± 77.95
**Midbrain**	WT	**257.06 ± 72.61**	**286.53 ± 39.60**	**144.62 ± 48.33**	148.54 ± 19.49	**95.56 ± 31.88**	**37.86 ± 14.62**	38.30 ± 14.39	703.00 ± 179.57
	Ube3a$^{(+/Pdup)}$	**154.34 ± 40.24**	**364.42 ± 54.73**	**299.88 ± 98.88**	145.95 ± 33.60	**58.01 ± 18.07**	**89.88 ± 33.94**	58.66 ± 15.56	839.67 ± 93.54
**Cortex**	WT	**902.23 ± 115.48**	**309.47 ± 73.39**	**226.40 ± 106.69**	**139.40 ± 39.05**	259.10 ± 39.92	106.83 ± 39.21	252.09 ± 62.00	507.00 ± 124.69
	Ube3a$^{(+/Pdup)}$	**663.49 ± 83.21**	**512.33 ± 135.20**	**360.37 ± 51.02**	**236.66 ± 64.68**	202.12 ± 40.68	147.29 ± 45.29	230.35 ± 749.13	286.33 ± 98.60
